# In search for an explanation to the upsurge in infant mortality in Kenya during the 1988–2003 period

**DOI:** 10.1186/1471-2458-12-441

**Published:** 2012-06-18

**Authors:** Sam W Wafula, Lawrence DE Ikamari, Boniface O K’Oyugi

**Affiliations:** 1 Population Studies and Research Institute, University of Nairobi, P.O. Box 30197–00100 GPO, Nairobi, Kenya

**Keywords:** Infant mortality, Decomposition, Malaria, HIV/AIDS, Kenya

## Abstract

**Background:**

In Kenya, infant mortality rate increased from 59 deaths per 1000 live births in 1988 to 78 deaths per 1000 live births by 2003. This was an increase of about 32 percent in 15 years. The reasons behind this upturn are poorly understood. This paper investigates the probable factors behind the upsurge in infant mortality in Kenya during the 1988–2003 period. Understanding the causes behind the upsurge is critical in designing high impact public health strategies for the acceleration of national and international public health goals such as the Millennium Development Goals (MDGs). The reversals in early child mortality is also regarded as one of the most important topics in contemporary demography.

**Methods:**

A merged dataset drawn from the Kenya Demographic and Health Surveys of 1993, 1998 and 2003 was used. The merged KDHS included a total of 5265 singletons. Permission to use the KDHS data was obtained from ICF international on the following website: http://www.measuredhs.com. Stata version 11.0 was used for data analysis. The paper used regression decomposition techniques as the main method for analysing the contribution of the selected covariates on the upsurge in infant mortality.

**Results:**

The duration of breastfeeding; maternal education, regional HIV prevalence and malaria endemicity were the factors that appeared to have contributed much to the observed rise in infant mortality in Kenya over the period. If all the live births that occurred in the 1996/03 period had the same mean values of all explanatory variables as those of live births that occurred in the 1988/95 period, then infant mortality would have increased by a massive 14 deaths per 1000 live births. However, if the live births that occurred in the 1988/95 period had the same mean values of all explanatory variables as those that occurred in the 1996/03 period, the upsurge in infant mortality would have been negligible. While the role of HIV in the upturn in infant mortality in Kenya and other sub Saharan African countries is indisputable, this study demonstrates that it is the duration of breastfeeding and Malaria endemicity that played a more significant role in Kenya’s upsurge in infant mortality during the 1988–2003 period.

**Conclusions:**

Efforts aimed at controlling and preventing malaria and HIV should be stepped up to avert an upsurge in infant mortality. There is need to step up alternative baby feeding practices among mothers who are HIV positive especially after the first six months of breastfeeding. Owing to the widely known inverse relationship between maternal education and infant mortality, there is need for concerted efforts to promote girl child education. Owing to the important role played by the short preceding birth interval to the upsurge in infant mortality, there is need to promote family planning methods in Kenya.

## Background

Despite having an average annual decline in infant mortality of about 4 per cent during the 1960–1980 period, [1,2]; Kenya experienced an upsurge in infant mortality during 1988–2003 period. Infant mortality rose from 59 per 1000 live births in 1988 to 78 per 1000 live births by 2003 [2]. This was an increase of about 32 percent during the 1988–2003 period. Recent studies have shown that the rise in infant mortality in Kenya over this period was not due to measurement errors or a deterioration in data quality [1,3]. Consequently, there must be other explanatory factors behind the upsurge in infant mortality. While literature is replete with the explanatory factors responsible for infant mortality decline in Kenya prior to the late 1980s, very little is known regarding the factors behind the upsurge in infant mortality during the 1988–2003 period [1,4,5].

An understanding of the reasons behind the upsurge in infant mortality in Kenya is critical and urgent in designing child survival programs and strategies. If clearly understood, priority interventions can be put in place for the quick realization of national and international targets such as target four under the Millennium development goals (MDGs). This paper had the following objectives: -

a) To examine the levels and trends in infant mortality during the 1988–2003 in Kenya

b) To identify the factors associated with the upturn in infant mortality in Kenya during the 1988–2003 period.

## Methods

### Sources of data

This paper used a merged data of the 1993, 1998 and 2003 Kenya Demographic and Health Surveys (KDHS). In total, 5265 singletons born five years preceding the 1993 and 2003 and three years before the 1998 KDHS were included in the study. The data were freely obtained from MEASURE DHS on the following website: http://www.measuredhs.com. We used the Mosley and Chen framework [6] to specify explanatory variables. This framework is the best for this study since it postulates that health can change if — and only if — one or more of the determinants change.

We used live births that occurred between 1988–95 to analyze infant mortality changes during the 1988–95 period and live births that occurred between 1996–2003 to analyze infant mortality changes during the 1996–2003 period. The dependent variable was the probability of dying between ages 0 and 11 months of life. This was a binary variable with 1 representing the occurrence of the event (i.e. infant death) during 0–11 months and 0 for censuring (i.e. for infants who never died). A detailed description of the Cox regression is available in any standard biostatistics textbook.

The following explanatory variables were used: the maternal age at child birth, the length of preceding birth interval, size of the baby at birth, duration of breastfeeding, source of water, type of toilet facility, maternal education, paternal education, ethnicity, household wealth index,^a^ place of delivery, whether the mother received a tetanus injection during pregnancy, HIV and malaria endemicity. These are some of the variables that were found to have changed the most during the 1988–2003 and hence were likely to be associated with the upsurge in infant mortality [6,7]. In the next section, we present a step by step description of our main analytical model - the regression decomposition technique.

### Application of the regression decomposition technique

In 1970s, Preston demonstrated that when demographic events such as infant mortality change, it is not only factors that determine such events that could be changing over time and space but rather, the events themselves could be changing at the same time as well [7]. This phenomenon makes traditional statistical techniques inappropriate because most of them assume that the dependant variable is constant. Regression decomposition techniques appear appropriate in such situations. These techniques have been widely used to study changes in mortality in developed countries and to a lesser extent developing countries [8-16].

In order to apply regression decomposition techniques, it is normally advisable that all selected explanatory variables are converted into dichotomous variables [5,9]. The dichotomization of the covariates is done based on theory and preliminary analysis of variables by the researcher to identify the specification that will produce the variables’ strongest effect on infant mortality. For instance, children of mothers who are young (below 20 years) or old (above 35 years) can be grouped together since such children would theoretically experience an elevated risk of infant mortality as compared to those born to mothers in the prime age (between 20–34 years) [10,11,16]. Below, we show how we categorized our explanatory variables into dichotomous variables with the exception of maternal and paternal education: -

a) Maternal age at birth (1 = Below 20 years & above 35 years 0 = 20–34 years)

b) Length of the preceding birth interval (0 < 24 months 1 = > 24 months)

c) Size of the baby at birth^b^(0 = Small 1 = Normal/Big)

d) Duration of breastfeeding (0 = Less than 10 months 1 > 10 Months)

e) Source of water (1 = Piped water 0 = Other)

f) Presence of toilet facility (1 = Yes 0 = No)

g) Maternal education: Years of schooling

h) Paternal education: Years of schooling

i) Ethnicity (1 = Kikuyu 0 = Other)

j) Household wealth status^c^(1 = Low 0 = High)

k) Place of delivery (1 = Health facility 0 = Home)

l) Had tetanus injection (1 = Yes 0 = No)

m) Period (0 =1988-95 1 = 1996/03)

n) Malaria endemicity^d^(0 = Low 1 = High)

o) Level of HIV prevalence^e^(0 = Low 1 = High)

The next step entails computing means of the dichotomized variables for each period while assessing whether the means are statistically different using a *t*-test statistic. In addition, it is usually important to establish the structure of the relationship between each explanatory variable and infant mortality in each period. Stata version 11.0 was used for statistical analysis. For purposes of accounting for censoring, Cox regression model was used to estimate the time of experiencing death between 0–11 months. The Cox regression provides the hazard ratios and the associated standard errors for each explanatory variable in the two periods under investigation [5,8,9]. In addition, a test of significance of the difference between the hazard ratio for each explanatory variable in the two periods was carried out in order to gauge whether the hazards were significantly different. The hazards were then used to account for the relative contribution of the differences in the structure of the relationship in explaining the difference in the level of infant mortality between two periods.

The critical question in using hazard ratios to predict the role of each explanatory variables on infant mortality between periods is: *Ceteris paribus,* what level of infant mortality would we have, say in the later period (1996/03) if a given explanatory variable in the initial period (1988/95) had the same mean value as in the later period (1996/03)? This question is repeated for each explanatory variable to assess their individual contribution to the observed upsurge in infant mortality [5,9]. This is illustrated in the example below:-

Assume that we would like to know the contribution of any covariate, say, household wealth Index (HWI) on infant mortality (IMR) in 1988/95 period if this variable values were applied to the 1996/03 period. We would have to use the following formula:-$\Delta \;in\;{\text{IMR}}_{0}=\text{β HWI}*{\text{IMR}}_{0}*\left(1-{\text{IMR}}_{0}\right)*\left({\text{HWI}}_{0}{-\text{HWI}}_{1}\right)*1000$

Where

Δ in IMR_0_ = change in infant mortality rate for the 1988/95 period

β HWI_0_ = the hazard ratio^f^ of HWI for the 1988/95 period

HWI_0_ = Mean value of the Household wealth Index for the 1988/95 period

β HWI_1_ = the hazard ratio of HWI for the 1996/03 period

HWI_1_ = Mean value of the Household wealth Index for the 1996/03 period

In the above formula, the equation is multiplied by 1000 to assess the contribution of the variable to infant mortality per 1000 live births.

## Results

### Trends in infant mortality by selected characteristics

Table 1 shows that while infants belonging to mothers in the 20–34 years had the lowest mortality rates as is conventionally expected, they experienced the highest increase (43 percent) during the study period. Further, the increase in the percentage of infant mortality among babies born with small body sizes was three times less as compared to the upsurge in infant deaths experienced among babies with average or large body sizes. Generally, shorter birth intervals are known to be associated with a heightened risk of early childhood mortality [1,3,5,9,10]. Results confirmed that infants with a preceding birth interval of <24 months experienced the highest upsurge in mortality (71 percent) during the 1989–2003 period.

**Table 1:** Levels and trends of infant and child mortality by selected characteristics

	1988-1995 *Cases =3115	1996-2003 *Cases = 2150	**% change 1988-2003**
**Mother’s Age**			
< 20 years	74.9	87.2	16.4
20- 34	51.4	73.6	**43.2**
35+	77.6	90.6	16.8
**Baby size at birth**			
Small	86.2	97.2	12.8
Av./Large	53.7	73.6	**37.1**
**Preceding Birth Interval**			
<24 months	76.7	130.8	**70.5**
25-36	51.5	72.5	40.8
37+	47.1	67	42.3
**Breastfeeding Period**			
<10 months	108.8	264.3	142.9
10-19 months	1.6	7.9	**393.8**
>20 months	92.9	1.5	−98.4
**Maternal education**			
None	59	95.2	**61.4**
Primary	69.5	85.4	22.9
Sec+	40.1	54	34.7
**Paternal Education**			
None	81.5	104.9	28.7
Primary	63.4	87.8	**38.5**
Sec+	47.1	61.2	29.9
**Ethnicity**			
Kalenjin	38.4	50.3	31.0
Kamba	43.1	75.9	76.1
Kikuyu	27.5	62.6	**127.6**
Kisii	64.1	37	−42.3
Luhya	53.5	92.3	72.5
Luo	142.9	79.5	−44.4
Other tribes	59.8	105.6	76.6
**Household wealth**			
Lower	42.1	75.6	**79.6**
Middle	56.5	80.1	41.8
Upper	79.4	62.3	−21.5
**Place of delivery**			
Home	68.8	83.4	21.2
Government Health facility	53.1	84	58.2
Private Health Facility	33.7	54.7	**62.3**
**Tetanus Toxoid**			
No	105.1	63.9	**−39.2**
Yes	54.4	53.6	−1.5

Notes: Primary analysis of merged KDHS 1993, 1998 & 2003. * Some variables do not add up to these figures because of missing cases. Preceding birth intervals include second and higher order births alone.

Source: Primary analysis of the merged KDHS dataset 1993–2003.

The duration of breastfeeding especially during the first six months of an infant has been found to improve infant survival due to the nutritional value of mother’s milk, the immunities against communicable diseases that it provides, and because it is hygienic [9]. Table 1 shows that infant mortality rose by 143 percent among infants who breastfed for less than 10 months during the study period. Infants belonging to mothers with no formal schooling experienced the highest upsurge in mortality (61 percent). Infants belonging to fathers with some primary education exhibited the highest upsurge in mortality (39 percent).

Infants belonging to minority ethnic groups categorized as “other”^g^ recorded an upsurge in infant mortality. This is likely due to the incessant inter-ethnic wars among this groups leading to displacement and death especially of women and children [16,17]. Likewise, infants belonging to Kikuyu women experienced a massive upsurge (128 percent) during the 1988–2003 study period. The exception to this trend was witnessed among the Kisii and Luo who recorded a decline in infant mortality rate during the study period (42 and 44 percent, respectively).

The results obtained indicate a decline in infant mortality among mothers belonging to the upper socio-economic class (22 percent) while those in the middle and lower tertiles experienced an upsurge in infant mortality during the 1988–95 and 1996–2003 period. Infant mortality rate among mothers who delivered at home rose by 21 percent. While being born in a government or a private health facility was associated with a lower risk of dying as compared to being born at home, generally, deliveries at a health facility recorded the highest upsurge in infant mortality rate (58 percent and 62 percent respectively). It is likely that there was an increase of high risk births delivered in health facilities.

Generally, infant mortality rate decreased among babies of mothers irrespective of whether such mothers had received tetanus injection or not. Among the infants born of mothers who had not received the tetanus injection, infant mortality decreased by 39 percent.

While trend analysis is insightful in bringing out the changes in infant mortality over the study period by selected characteristics, it fails to show whether the observed changes are statistically significant. We address this gap in the next section.

### Explanatory variables of infant mortality between the two periods

Results from Table 2 show that the mean values for the duration of breastfeeding were significantly different between the two periods. Other variables that were significantly different between the two periods were maternal education, regional HIV prevalence and regional malaria endemicity. These results imply that the observed changes in infant mortality in Kenya during the 1988–95 and 1996–2003 periods could be partly accounted for by the above four variables.

**Table 2:** Means values of the explanatory variables for infant mortality: Kenya, 1988–95 and 1996–2003 periods

**Characteristic**	**Time period**	**Time period**	**Significance of differences in means (t-statistic)**
	**1988–95 (n = 3115)**	**1996–03 (n = 2150)**	
**Maternal factors**			
Maternal age at child birth (D)	1.672	1.688	−0.951
Baby size at birth (D)	0.818	0.816	0.175
Duration of breastfeeding (D)	0.810	0.908	−7.525**
Preceding birth interval (D)	0.699	0.736	−1.93
**Maternal health-seeking factors**			
Place of delivery (D)	0.624	0.593	1.43
Tetanus Toxoid (D)	0.883	0.867	1.297
**Socio-economic factors**			
Paternal education (years)	5.471	5.789	−1.141
Source of water (D)	0.268	0.299	−1.746
Type of Toilet Facility (D)	0.795	0.812	−1.145
Maternal education (Years)	5.149	5.490	−4.068**
Ethnicity (D)	0.867	0.882	−1.248
Household Wealth Index (D)	0.558	0.580	−1.92
**Community level factors**			
HIV regional status (D)	1.364	1.429	3.388**
Malaria endemic region (D)	0.345	0.421	−4.344**

Notes: D denotes dichotomous variable. ***p < 0.001, **p < 0.01, *p < 0.05.

Source: Primary analysis of the merged KDHS dataset 1988/89 and 1997/98.

Below, we assess the role of the explanatory variables in relation to the rising levels of infant mortality between 1988–95 and 1996–2003 periods.

### Effects of the explanatory variables on observed infant mortality in the two periods

Table 3 shows the results of Cox regression models. In Model I the risk of dying in infancy during the 1996–2003 period was 19 percent higher as compared to the risk of dying during the 1988–95 period. When maternal factors were added to the model as shown in Model II, the risk of dying in infancy during the 1996–2003 was only 3 percent higher as compared to the 1988–95 period although this association was not statistically significant (p-value > 0.05). However, in Model III when the socio-economic factors were added to Model I, the risk of dying in infancy was 23 percent higher during the 1996–2003 as compared to the 1988–1995 period and this increase was statistically significant (p-value < 0.05). Likewise, in Model IV when health seeking behavior factors were added to Model I, the risk of dying in infancy in 1996–2003 was 10 percent higher than in 1988–1995 and this risk was statistically significant (p < 0.05).

**Table 3:** Hazard Ratios and likelihood chi-square values indicating the effects of period of birth controlling for explanatory variables on infant mortality, merged KDHS 1993-2003

**Explanatory variable**	**Model I**	**Model II**	**Model III**	**Model IV**	**Model V**	**Model VI**
LRχ2	7.18	33.07	64.3	38.19	110.59	102.57
d.f.	1	1	1	1	1	1
P-value	< 0.001	< 0.001	< 0.001	< 0.001	< 0.001	< 0.001
Time period						
1988-95	1.0	1.0	1.0	1.0	1.0	1.0
1996-2003	1.19 (2.69)*	1.03 (0.35)	1.23 (2.42)*	1.10(5.17)*	1.28(2.46)*	1.38(2.41)*

**Notes:** Model 1 = Time period indicator only, Model II = Model I plus all maternal factors, Model III = Model I plus all socio-economic factors (SES); Model IV = Model I plus all maternal health seeking factors; Model V = Model I plus community level factors and Model VI = Model I plus all other models. T-statistics are shown in the parentheses; *significant at p-value < 0.05. The LRχ2 values refer to those associated with the time period indicator variable.

Source: Primary analysis of the pooled KDHS Datasets, 1993, 1998 & 2003.

In Model V when the regional HIV prevalence and Malaria variables were added to Model 1, the risk of dying in the 1996–2003 period was 28 percent higher than that of the 1988–95 period (p-value < 0.05). Finally, when the full model was considered, the risk of dying in 1996–2003 was 38 percent higher as compared to the 1988–95 period and this association was statistically significant (p < 0.05).

As shown in Table 3, when all explanatory variables were added to Model I, the likelihood ratio test satisfied p < 0.001 for all the six models. This implies that the explanatory power of the additional variables more than compensated for the loss in degrees of freedom. Generally, the results above imply that the differences in the values of explanatory variables, taken together, during the two periods contributed to the differences in the observed infant mortality. In the next section, we assess the contribution of these variables on infant mortality changes by first assuming a fixed structure analysis and then a changing structure between the variables and infant mortality.

### Structure of relationships in the two periods

As discussed earlier, infant mortality is bound to have changed independent of the explanatory variables considered in this study [7]. It is thus important to factor in this analysis the role of the structure of infant mortality *vis a vis* explanatory variables in the observed upsurge in infant mortality. This section and the subsequent sections address this issue by first assuming a fixed structure analysis and later, a changing structure analysis. Table 4 presents the regression coefficients (log odds) of the explanatory variables during the two periods. Column C of Table 4 shows the results of the test of significance of the coefficients between the two periods. In the 1988–95 period, baby size, duration of breastfeeding, the preceding birth interval, household wealth index, regional HIV status and regional Malaria endemicity status had each a significant effect on infant mortality. However, in 1996–03 period it is only the baby size, duration of breastfeeding, malaria endemicity and regional HIV prevalence that had each a significant effect on infant mortality.

**Table 4:** Coefficients of explanatory variables of infant mortality between 1988/95 and 1996/2003, A pooled KDHS Dataset

**Characteristic**	**Coefficients**	**Coefficients**	**Significance of differences in coefficients for the two periods (t-statistic) (c)**
	**period (1988–95) (n = 3115) (a)**	**period (1996–03) (n = 2150) (b)**	
**Maternal factors**			
Maternal age at child birth (D)	−0.430 (−1.02)	0.035 (0.08)	−0.114
Baby size at birth (D)	−0.456 (−2.01)***	−0.367 (−2.40)***	5.897***
Duration of breastfeeding (D)	1.032 (5.82)**	4.179 (8.12)***	6.969***
Preceding birth interval (D)	−0.402 (−2.29)**	−0.589 (−1.72)	6.094***
**Maternal health-seeking factors**			
Place of delivery (D)	0.228 (1.16)	0.103 (0.47)	2.624*
Tetanus Toxoid (D)	−0.423 (−1.56)	−0.129 (−0.40)	0.864
**Socio-economic factors**			
Paternal education (years)	−0.052 (−1.19)	0.015(0.77)	−64.717***
Source of water (D)	0.066 (0.260)	−0.392(−1.30)	−19.901***
Type of Toilet Facility (D)	−0.399 (−1.85)	0.243(0.82)	−2.316**
Maternal education (Years)	−0.040 (−0.94)	−0.017(−0.38)	56.965***
Ethnicity (D)	−0.192(−0.54)	0.092 (0.22)	−0.831
Household Wealth Index (D)	−0.497(−2.37)**	−0.167 (−0.72)	1.757
**Community level factors**			
HIV regional status (D)	1.082 (5.15)***	0.933 (3.86)***	4.289***
Malaria endemic region (D)	−1.265(−5.06)***	−1.332 (−4.78)***	3.831***

Notes: D denotes dichotomous variable. ***p < 0.001, **p < 0.01, *p < 0.05. Column C was computed using the following formula: (P_2_-P_1_)/(1.96SQRT(X_1_ + *X*_2_) where P_2_& P_1_are the coefficients of the two periods and X_1_& X_2_are the standard errors for each explanatory variable for the two periods.

Source: Primary analysis of the merged KDHS dataset 1993–2003.

The effect of the baby size at birth was much greater during the 1996–03 period than in 1988–95 period. The effect of duration of breastfeeding was more pronounced during the 1988–95 than in the 1996–03 period. Duration of breastfeeding and regional HIV status had each a positive significant effect on infant mortality. The effect of duration of breastfeeding on infant mortality was however greater during the 1996/03 period than during the 1988–95 period. On the other hand, the effect of HIV seemed to have been more pronounced during the 1988–95 period as compared to the 1996/03 period. The effect of HIV regional prevalence and maternal education was positive and significant while that of paternal education, source of water and type of toilet facility was significant but negative.

### Explanatory variables versus structural relationships

The preceding section presumed a fixed structure analysis (i.e. that it is only the covariates that changed over time and not infant mortality). This section introduces a changing structural analysis (i.e. that apart from covariates, infant mortality could have changed as well). Table 5 shows that the observed level of infant mortality between the 1988–95 period was 61.3 deaths per 1000 live births against 69.6 deaths per 1000 live births for the 1996–03 period. This means that the difference in the observed level of infant mortality between the two periods was 8.3 deaths per 1000 live births.

**Table 5:** Changes in infant mortality rate had each time period experienced the same value of explanatory variables

**Characteristic**	**Mean values for 1988-95**	**Mean values for 1996-03**	**β’s for 1988-95**	**β’s for 1996-03**	**Change in IMR if 1988/95 had the same mean value of this variable as the 1996/03 period**	**Change in IMR if 1996/03 had the same mean value of this variable as the 1988/95 time period**
**Maternal factors**						
Maternal age at child birth (D)	1.672	1.688	0.011	−0.077	−0.010	−0.080
Baby size at birth (D)	0.818	0.816	−0.122	−0.0568	−0.014	0.007
Duration of breastfeeding (D)	0.81	0.908	0.858	3.053	−4.838	19.375
Preceding birth interval (D)	0.699	0.736	−0.294	−0.373	0.626	−0.894
**Maternal health-seeking behaviour**						
Place of delivery (D)	0.624	0.593	0.284	0.207	0.507	−0.416
Tetanus Toxoid (D)	0.883	0.867	−0.251	−0.174	−0.231	0.180
**Socio-economic factors**						
Paternal education (years)	5.471	5.789	0.005	0.011	−0.091	0.227
Source of water (D)	0.268	0.299	0.075	−0.31	−0.134	−0.622
Type of Toilet Facility (D)	0.795	0.812	−0.399	0.082	0.390	0.090
Maternal education (Years)	5.149	5.49	−0.057	−0.019	1.118	−0.420
Ethnicity (D)	0.867	0.882	−0.146	0.096	0.126	0.093
Household Wealth Index (D)	0.558	0.58	−0.373	−0.177	0.472	−0.252
**Community level factors**						
HIV regional status (D)	1.364	1.429	0.829	0.899	−3.101	3.784
Malaria endemic region (D)	0.345	0.421	−1.21	−1.405	5.292	−6.915
**Sum**					**0.112**	**14.159**
IMR	0.0613	0.0696				
P*(1-P)	0.0575	0.0648				

Notes: D denotes dichotomous variable. Source: Primary analysis of the merged KDHS dataset 1993–2003.

Source: Primary analysis of the merged KDHS dataset 1993–2003.

In general, the results shown in Table 5 indicate that if all the infants that were born during the 1996–03 period had the same mean values of all explanatory variables as those of 1988–95 period, then infant mortality would have increased by a massive 14 deaths per 1000 live births. However, had infants that occurred in the 1988/95 period had the same mean values of all explanatory variables as those that occurred in the 1996–03 period, the upsurge in infant mortality could have been negligible.

In particular, duration of breast feeding, preceding birth interval, the regional level of HIV prevalence and regional Malaria prevalence appear to account for the largest share of the differences in mortality between the two periods.^h^Had infants belonging to the 1996–03 period experienced the same mean values of duration of breastfeeding as those belonging to the 1988–95 period, infant mortality could have increased by a massive 19 deaths per 1000 live births. In contrast, had infants belonging to mothers in 1988–95 period experienced the same mean values of duration of breast feeding as those of children belonging to mothers in the 1996–03 period, infant mortality would have reduced by 5 deaths per 1000 live births

Regarding malaria endemicity, had infants belonging to period 1996–03 experienced the same mean values of the disease as those belonging to the 1988–95 period, then infant mortality would have reduced by 7 deaths per 1000 live births. In the case of HIV/AIDS, the reduction would have been 3 deaths per 1000 live births. However, had infants belonging to the 1988–95 period experienced the same mean values of malaria endemicity and HIV/AIDS as those of belonging to the 1996–03 period, infant mortality would have increased by 5 deaths and 4 deaths per 1000 live births, respectively. The results also seem to indicate that if the rest of the explanatory variables for the 1996–03 period had values as those born in the 1988–95 period, then infant mortality rate in the 1996/03 would have remained largely unaltered and vice versa.

### Discussion, conclusion and recommendations

This paper sought to explain the upturn in infant mortality in Kenya during the 1988–2003 period using regression decomposition techniques. The findings revealed that apart from maternal age at child birth, tetanus toxoid, ethnicity and household wealth index, the effect of the rest of the explanatory variables were significantly associated with explaining the differences in infant mortality over the study period. In particular, the duration of breastfeeding, maternal education, regional HIV and malaria prevalence contributed much to the observed rise in infant mortality. However, there could be other important factors behind the upsurge that were not captured by KDHS. For instance, KDHS does not collect information on injury related factors yet an upsurge in these factors could have influenced the upturn in infant mortality during the study period.

The above findings have several implications. First, there is need for the urgent promotion of family planning services in order to reduce the adverse effect of short birth intervals as well as prevent high risk births which tend to be associated with a heightened risk of infant deaths [5,11]. Second, in a generalized HIV setting like Kenya, there is need for the government with the assistance of developing partners to strengthen the prevention of mother to child (PMTCT) programs in order to eliminate new pediatric HIV infections especially in high HIV prevalence regions. This may entail promoting exclusive breastfeeding during the first six months among HIV positive mothers and weaning infants thereafter by introducing alternative feeding options as well as encouraging family planning integration in PMTCT programming to postpone or limit births. Through this, fewer babies will be born with low birth weight, which is a risk factor for mortality.

Household wealth index- a proxy measure of the socio-economic status of the economy played an increasing role in infant mortality upturn. The downturns in Kenya’s economy during the study period with some years recording a negative growth in the GDP must have depressed the household level spending on health. This was further worsened by the introduction of the structural adjustment programs (SAPs) in the late 1980s which among other things included cost sharing in health and education and was also accompanied by job cuts in the public sector [18,19]. There is need for an increase in budgetary allocation to health to meet international targets such as the allocation of 15 percent of the annual budget on health as was passed in the Abuja Declaration of 2000. The increases in budgetary allocation will cover substantially the health provision to the majority poor who currently cannot afford any form of healthcare.

Findings show that malaria prevalence played an important role in the observed upturn in infant mortality. This was expected owing to the fact that malaria resistance peaked in Kenya in 1997 (when resistance to chloroquine ranged from 66–87 percent). This forced the government to change the first line of treatment for malaria from chloroquine to artemisinin combination therapy (ACT) for management treatment of uncomplicated Malaria cases [18,20]. There is need for intensified use of anti malaria prophylaxis among pregnant women and children as well as early diagnosis and treatment of Malaria. Additionally, programs aimed at intensifying the distribution of insecticide-treated mosquito nets, in-house spraying and other vector control strategies in high Malaria-prone regions as well as biomedical research for malaria vaccine development need renewed emphasis in Kenya’s health programming.

The deterioration in gross enrolment rates in education especially after the introduction of SAPs in the late 1980s appears to have locked out many young girls in the education system. There is need for renewed efforts to promote the girl child education owing to the widely known role of maternal education in reducing infant mortality [9,11,16].

Unlike past studies [1,3] this study sheds more light on this topic by employing a more robust methodological approach. It also brings on board important but under researched factors such as breastfeeding, environmental conditions (such as sanitation, potable water) and improved quality of and access to health care that were not considered in recent studies that employed regression decomposition techniques [10]. The key results here show that far from the widely cited important role of HIV in the upturn in infant mortality in Kenya and other sub Saharan African countries [1,3], the duration of breastfeeding and Malaria endemicity played a more significant role in Kenya’s upsurge in infant mortality during the 1988–2003 period.

## End Notes

^a ^We computed the household wealth index using the principle component analysis (PCA). The first principle component was used to compute household tertiles i.e. low, middle and high income households.

^b^ Conventionally, infants born with a birth weight of less than 2500grams are categorized as low birth weight (LBW) and vice versa. While our study did not use this variable due to large number of missing cases (55%) arising from low health facility deliveries in Kenya (only 42%), we used baby size as a proxy measure of low birth weight.

^c^ Household wealth index was computed using the principle component analysis (PCA) based on the household possessions of each sampled household. Those with low and medium income were categorized as “low” while those with high income were categorized as “high.” In our preliminary analysis, we found that low and medium households experienced an upsurge in mortality while the high income households experienced a decline in mortality.

^d^ The categorization of whether a region was low or high malaria endemicity was based on the categorization of Kenya Medical and Research Institute which was used in one of the author’s earlier study [5]. Accordingly, Nyanza, Western, Coast and North Eastern provinces were categorised as high malaria endemic regions while Central, Eastern, Nairobi and Rift valley were categorized as low malaria endemic regions.

^e^ This paper categorized regions with higher than national HIV prevalence (6.7%) as high HIV prevalence regions and vice versa [2]. Using this categorization, Nyanza (18.3%); Central (7.6%); Nairobi (11.9%); Rift Valley (6.9%) were grouped as high HIV prevalence while Western (5.8%); Coast (6.6%); Eastern (6.2%) and North Eastern (0.0%) were categorized as low HIV prevalence.

^f^ For ease of interpretation, the hazard ratios were exponentiated to give regression coefficients (i.e. exp(HR) = β) used as shown in Tables 2 and 3.

^g^ Ethnic groups categorized as “other” included all tribes apart from the Kikuyu. These include the Kalenjin, Luhya, Luo, Kisii, Kamba and other small tribes such as the Somali, Rendile, Pokomo, TaitaTaveta, Borana, etc.

^h^ Although our decomposition analysis focuses on the relative importance of maternal, socio-economic, and health seeking factors on the change in infant mortality over this time period, our study does not examine the important interrelationships occurring between these groups of variables. For instance, maternal education is widely known to be related with age at birth as well as maternal health seeking behavior. It is beyond the scope of this paper to disentangle whether changes in socio-economic factors are causing changes in maternal and health seeking factors or the other way round or if their simultaneous changes are caused by another factor.

## Competing interests

The authors affirm they have no competing interests.

## Authors’ contributions

SWW wrote the draft paper which is based on a chapter of his PhD dissertation. LI and BK reviewed the paper. All the three authors approved the manuscript prior to submission.

## Pre-publication history

The pre-publication history for this paper can be accessed here:

http://www.biomedcentral.com/1471-2458/12/441/prepub
